# Identification of two major QTLs for pod shell thickness in peanut (*Arachis hypogaea* L.) using BSA-seq analysis

**DOI:** 10.1186/s12864-024-10005-x

**Published:** 2024-01-16

**Authors:** Hongfei Liu, Zheng Zheng, Ziqi Sun, Feiyan Qi, Juan Wang, Mengmeng Wang, Wenzhao Dong, Kailu Cui, Mingbo Zhao, Xiao Wang, Meng Zhang, Xiaohui Wu, Yue Wu, Dandan Luo, Bingyan Huang, Zhongxin Zhang, Gangqiang Cao, Xinyou Zhang

**Affiliations:** 1 Institute of Crop Molecular Breeding, The Shennong Laboraory, Key Laboratory of Oil Crops in Huang-Huai-Hai Plains, Henan Provincial Key Laboratory for Oil Crops Improvement, Postgraduate T&R Base of Zhengzhou University, Henan Academy of Agricultural Sciences, Ministry of Agriculture, Zhengzhou, 450002 China; 2 School of Agricultural Sciences, Zhengzhou University, Zhengzhou, 450002 China; 3 School of Life Sciences, Zhengzhou University, Zhengzhou, 450002 China

**Keywords:** Peanut, Pod shell thickness (PST), Bulked sergeant analysis sequencing (BSA-seq), Quantitative trait locus (QTL), Kompetitive allele-specific PCR (KASP), Fine mapping

## Abstract

**Background:**

Pod shell thickness (PST) is an important agronomic trait of peanut because it affects the ability of shells to resist pest infestations and pathogen attacks, while also influencing the peanut shelling process. However, very few studies have explored the genetic basis of PST.

**Results:**

An F_2_ segregating population derived from a cross between the thick-shelled cultivar Yueyou 18 (YY18) and the thin-shelled cultivar Weihua 8 (WH8) was used to identify the quantitative trait loci (QTLs) for PST. On the basis of a bulked segregant analysis sequencing (BSA-seq), four QTLs were preliminarily mapped to chromosomes 3, 8, 13, and 18. Using the genome resequencing data of YY18 and WH8, 22 kompetitive allele-specific PCR (KASP) markers were designed for the genotyping of the F_2_ population. Two major QTLs (*qPSTA08* and *qPSTA18*) were identified and finely mapped, with *qPSTA08* detected on chromosome 8 (0.69-Mb physical genomic region) and *qPSTA18* detected on chromosome 18 (0.15-Mb physical genomic region). Moreover, *qPSTA08* and *qPSTA18* explained 31.1–32.3% and 16.7–16.8% of the phenotypic variation, respectively. Fifteen genes were detected in the two candidate regions, including three genes with nonsynonymous mutations in the exon region. Two molecular markers (Tif2_A08_31713024 and Tif2_A18_7198124) that were developed for the two major QTL regions effectively distinguished between thick-shelled and thin-shelled materials. Subsequently, the two markers were validated in four F_2:3_ lines selected.

**Conclusions:**

The QTLs identified and molecular markers developed in this study may lay the foundation for breeding cultivars with a shell thickness suitable for mechanized peanut shelling.

**Supplementary Information:**

The online version contains supplementary material available at 10.1186/s12864-024-10005-x.

## Background

Cultivated peanut (*Arachis hypogaea* L.; 2n = 4x = 40) is an important oil and cash crop that is grown worldwide, with an annual global yield of about 54 million tons and harvest area of over 32 ha (FAOSTAT, http://www.fao.org/faostat/en/#data/QC, 2021). Because of their unique biological characteristics, peanuts must be shelled before they can be used (e.g., oil extraction, food processing, and seeding). Shelling refers to a process that breaks the peanut shell and separates it from the kernel [1, 2].

Pod shell thickness (PST), which is calculated as the distance between the exocarp and the mesocarp of peanut, is an important peanut shell trait that affects peanut processing and resistance to pest infestations [3]. Thick-shelled peanuts are suitable for storage, but breaking and removing their shells can be difficult (i.e., decreased shelling efficiency), whereas thin-shelled peanuts are appropriate for shelling, but they are susceptible to pest infestations and their seeds may be damaged during shelling [4, 5]. Therefore, optimizing PST is conducive to maximizing the mechanized shelling efficiency and peanut quality, while also improving peanut storage and processing, potentially leading to increases in the economic value of peanuts.

A previous study revealed that PST is a complex quantitative trait with a normal distribution [3]. Pod shell thickness is typically determined by measuring the thickness of the pod waist [6], the ridge of the pod posterior chamber [7, 8], the pod stalk [9], and the most convex part of the posterior chamber [3] using a digital vernier caliper. Guo et al. were the first to use vernier calipers to measure the mature pod waist and calculate the shell thickness. Moreover, they used 204 chromosome segment substitution lines obtained from a cross between the recurrent parent Qinhuangdaoguangyang and the donor parent Shiyaodou to identify QTLs for PST, among which two QTLs were detected on chromosomes 2 and 15, with phenotypic variance explained (PVE) values of 7.65% and 9.00%, respectively [6]. Li et al. used a recombinant inbred line (RIL) population comprising 151 lines derived from 79,266 and D893 to identify QTLs for PST; 14 QTLs were detected in seven environments (PVE value of 6.90–23.16%), while *qPST12* on chromosome 12 was detected in three environments (PVE value greater than 20%) [7]. Liu et al. analyzed a RIL population consisting of 441 lines derived from Shanhua15 and Zhonghua12 and identified four QTLs on chromosomes 7, 8, 13, and 17 (PVE value of 1.75–3.22%) [8]. Yang et al. examined the 267 lines of a RIL population derived from Yueyou92 and Xinhuixiaoli and detected three QTLs on chromosomes 4, 7, and 13 (PVE of 7.36–11.61%) [9]. In a recent study, one stable major QTL for PST (*qAHPS07*) was finely mapped to a 36.46-kb physical interval on chromosome 7 [10]. However, PST measurements are generally imprecise and the complex genetic mechanisms underlying this trait remain relatively uncharacterized.

A bulked segregant analysis (BSA), which involves the pooling of samples with extreme phenotypes, may be useful for cost-effective and highly efficient marker-based screening or QTL mapping [11]. During the last decade, advances in next-generation sequencing technologies and decreases in sequencing costs have enabled researchers to gradually develop and improve BSA sequencing (BSA-seq) methods and technical systems for analyses of multiple crops, including maize [12], soybean [13], rice [14], and wheat [15]. They have also been used in studies on various peanut traits, including testa color [16], branch number [17], sucrose content [18], and pod size [10].

Four kinds of statistical algorithms can be used to identify the single nucleotide polymorphisms (SNPs) or genomic regions associated with target traits by BSA-seq analysis. The SNP-index is a classical algorithm. Specifically, Δ(SNP-index) is used to calculate the difference in the genotype frequency between mixed pools. A strong association between the genomic region and the target trait is reflected by a Δ(SNP-index) value close to 1 [19]. The Euclidean distance (ED) algorithm is used to identify the SNP sites that differ significantly between two mixed pools as well as to evaluate the regions associated with traits. Theoretically, in the two mixed pools constructed for a BSA, all loci except for those related to the target trait tend to be the same. Thus, the ED of non-target loci should be close to 0. The magnitude of ED represents the degree of the difference in the markers between the two mixed pools [20]. The *G* value is a modified statistic obtained after smoothing the *G* statistic, which is useful for mapping a relatively narrow region. The *G* value of each SNP is calculated on the basis of the allele sequencing depth, and is weighted according to the physical distance of the adjacent SNP [21]. Fisher’s exact tests were used to calculate *P* values at each variant position to generate a *P* value-based plot corresponding to the genomic position [22].

Kompetitive allele-specific PCR (KASP) is a fluorescence-based genotyping technology that enables the accurate detection of bi-allelic SNP and insertion-deletion (InDel) sequences at specific loci in complex genomic DNA samples [23]. It is potentially very useful for improving crop traits because of its high flux and strong operability [24]. In addition, BSA-seq may be combined with KASP marker-based fine mapping for the rapid and efficient identification of QTLs linked to target traits. This combined approach has been widely applied to identify QTLs for agronomic traits in diverse crops, including plant height in rice [25], powdery mildew resistance in melon [26], kernel length in wheat [27], and sucrose content in peanut [18].

In this study, a new method for calculating PST was proposed and then BSA-seq and fine mapping were used to identify QTLs for PST in an F_2_ population obtained from a cross between Yueyou 18 (YY18) and Weihua 8 (WH8). The results of this study may be used to further clarify the genetic mechanism underlying PST, while also providing the theoretical basis for developing relevant molecular markers and cloning important genes.

## Results

### Phenotypic identification of the two parents and the F_2_ population

**Fig. 1:**
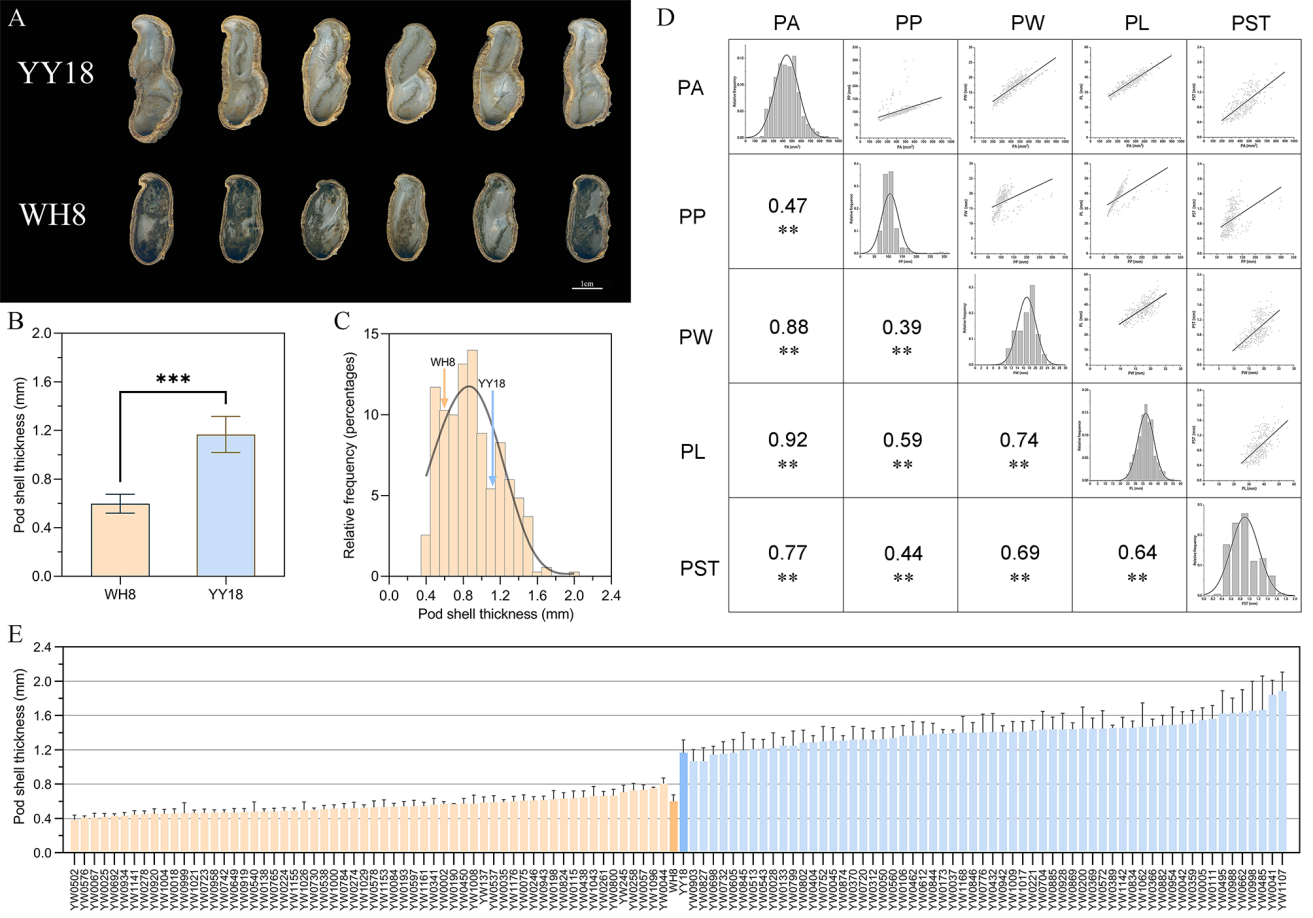
Phenotypic analysis of the two parents and the F_2_ population. **(A)** Phenotypes of the mature pod shells from YY18 and WH8. Bar = 1 cm. **(B)** Phenotypic difference between YY18 and WH8 in pod thickness. **(C)** Frequency distribution of PST in the 410 individuals of the F_2_ population. **(D**) Correlation analysis of the pod size-related traits in the 350 individuals of the F_2_ population. **(E)** Data for the pod shell thickness of each F_2_ individual selected to construct the mixed bulks. **, *P* < 0.01; ***, *P* < 0.001

The phenotypic analysis revealed significant differences in PST between YY18 and WH8 (Fig. 1B). The continuous variation in the PST of 410 selected individuals was consistent with an approximately normal distribution (Fig. 1C, Table S1), with maximum and minimum values of 1.96 and 0.36 mm, respectively, and a coefficient of variation of 34.7% (Table S2). The correlation analysis of 350 materials indicated PST was significantly positively associated with the pod area (PA), pod perimeter (PP), pod length (PL), and pod width (PW) (Fig. 1D). The broad-sense heritability of the pod shell thickness, pod area, pod perimeter, pod width and pod height was 0.9801, 0.9213, 0.9571,0.8746 and 0.9136, respectively. One-way analysis of variance showed that PST was reproducible in one material and varied significantly in different materials (Table S3).

### BSA-seq analysis of PST

A total of 399.87 Gb raw data were generated by the sequencing platform. After filtering for quality, 398.06 Gb clean data were retained, with an average of 99.52 Gb per sample. The reads with Q20 value ≥ 95.40%, Q30 value ≥ 88.15%, and error rate ≤ 0.04 were used for the subsequent variant detection and analysis. The mean genome coverage depth was 43.66×, 42.52×, 85.32×, and 83.03× for YY18, WH8, thick-shelled pool, and thin-shelled pool, respectively. Additionally, 99.80%, 98.25%, 98.91%, and 98.82% of the clean reads for YY18, WH8, thick-shelled pool, and thin-shelled pool, respectively, were mapped to the reference genome (Table 1). A total of 351,510 high-quality homozygous SNPs/InDels between the thick-shelled pool and the thin-shelled pool were obtained after removing the low-quality SNPs and InDels as well as the heterozygous sites in the two parents. These SNPs/InDels were used to conduct the association analysis for PST.

**Table 1:** The summary of the sequencing quality and alignment results for BSA-seq analysis

Sample ID	Raw Base (bp)	Clean Base (bp)	Effective Rate (%)	Error Rate (%)	Q20 (%)	Q30 (%)	GC Rate (%)	Mapped (%)	Coverage Rate (%)	Mean Depth
Thin-pool	173,104,773,300	172,337,628,000	99.56	0.04	95.19	88.15	36.61	99.74	98.82	83.03×
Thick-pool	168,069,016,500	167,308,148,700	99.55	0.03	95.94	89.66	36.86	99.76	98.91	85.32×
WH8	28,116,614,100	27,991,626,600	99.56	0.03	95.5	88.75	36.51	99.65	98.25	42.52×
YY18	30,575,037,300	30,427,013,700	99.52	0.04	95.4	88.59	36.37	99.8	98.62	43.66×

Sample ID, name of sample; Raw Base (bp), number of original bases; Clean Base (bp), number of filtered bases; Effective Rate (%), proportion of the raw bases that were clean bases; Error Rate (%), proportion of all bases that were incorrect; Q20 (%), proportion of all bases with a Phred value greater than 20; Q30 (%), proportion of all bases with a Phred value greater than 20; GC Rate (%), proportion of all bases that were C or G; Mapped (%), proportion of the clean bases that were mapped to the reference genome; Coverage Rate (%), proportion of the genome covered by the bases; Mean Depth, average sequencing depth

**Fig. 2:**
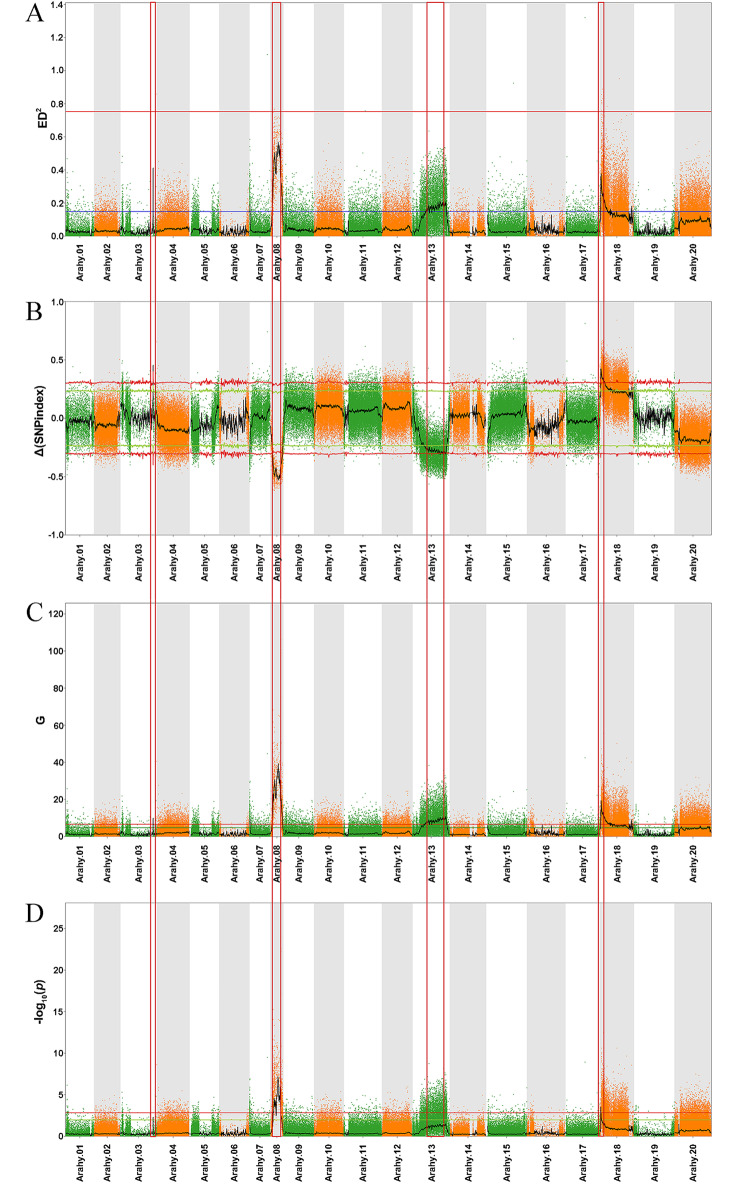
QTL analysis of the pod shell thickness based on BSA-seq. Manhattan plots were generated to present the squared ED **(A)** and the distribution of Δ(SNP-index) **(B)**, the distribution of *G* values **(C)**, and the distribution of - log_10_(*P* values) according to Fisher’s exact test **(D)** on chromosomes. The green/blue and red lines represent 95% and 99% confidence intervals, respectively. The black lines indicate the average values for the four algorithms based on the sliding windows analysis. The significant genomic regions related to PST are indicated by red rectangles

The Δ(SNP-index), ED, *G* value, and Fisher’s exact test data were plotted according to the chromosomal distribution of each parameter (Fig. 2). The overlapping intervals among the four methods were mapped to a 2.0-Mb region (*Arahy.03*: 127,500,001–129,500,000 bp), an 18.17-Mb region (*Arahy.08*: 22,920,001–41,090,000 bp), a 6.70-Mb region (*Arahy.13*: 125,790,001–132,490,000 bp), and a 12.87-Mb region (*Arahy.18*: 3,490,001–16,360,000 bp) on chromosomes 3, 8, 13, and 18, respectively (Table 2).

**Table 2:** The candidate regions associated with pod shell thickness based on BSA-seq analysis

Chromosome	Start (bp)	End (bp)	Size (Mb)	Direction	Peak	Algorithm
Arahy.03	127,500,001	129,500,000	2.00	upper	0.4533333/0.4110222	Δ (SNP-index) /ED
Arahy.08	22,920,001	41,090,000	18.17	lower	-0.5320390/0.0000432	Δ (SNP-index) /*G* value
Arahy.13	125,790,001	132,490,000	6.70	lower	-0.3104813	ED
Arahy.18	3,490,001	16,360,000	12.87	upper	0.4254386	ED

### KASP marker development and fine mapping for PST

**Fig. 3:**
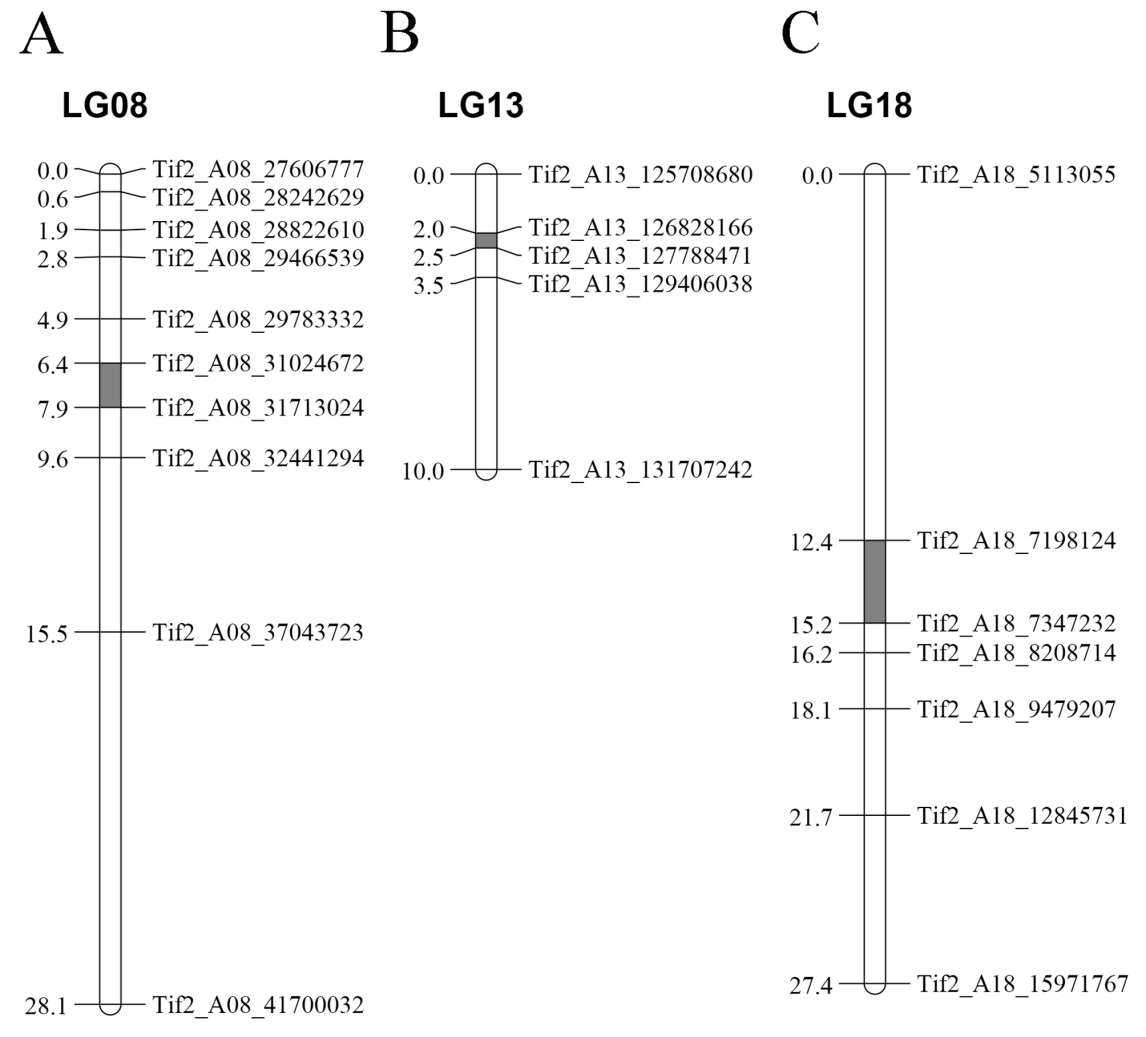
Fine mapping of QTLs related to pod shell thickness. Fine mapping of QTLs related to pod shell thickness on chromosomes 8 **(A)**, 13 **(B)**, and 18 **(C)**. The identified QTLs are indicated by gray boxes

To narrow down the candidate regions, 10, 5, and 7 KASP markers were designed for the candidate regions on chromosomes 8, 13, and 18, respectively (Table S4). The 410 individuals randomly selected from the F_2_ population were genotyped using the 22 KASP markers and then the genetic linkage analysis was performed. The results revealed that the genomic region on chromosome 3 detected by BSA-seq was not identified during the linkage analysis, indicative of a false positive locus. The BSA-seq and linkage analysis suggested that three QTLs (*qPSTA08*, *qPSTA13*, and *qPSTA18*) were associated with PST. The major QTL *qPSTA08*, which was detected on chromosome 8, had PVE and LOD values of 31.3–32.3% and 27.5–28.7, respectively, with a genetic distance of 1.6 cM and a physical distance of 0.69 Mb (31,024,672–31,713,024 bp) (Fig. 3A). In contrast, *qPSTA18*, which was detected on chromosome 18 in a region highly homologous to *qPSTA08*, had PVE and LOD values of 16.7–16.8% and 13.5–13.6, respectively, with a genetic distance of 2.83 cM and a physical distance of 0.15 Mb (7,198,124–7,347,232 bp) (Fig. 3C). Moreover, the *qPSTA18* genome sequence was highly homologous to part of the *qPSTA08* genome sequence. The minor QTL (*qPSTA13*), which was mapped to a 0.96-Mb physical interval (126,828,166–127,788,471 bp) on chromosome 13, had a PVE value of 6.4–6.6% (Fig. 3B; Table 3).

**Table 3:** The QTLs associated with pod shell thickness identified by fine mapping

Chromosome	Start (bp)	End (bp)	Size (Mb)	Interval (cM)	LOD	PVE (%)	ADD (%)	Dom
Arahy.08	31,024,672	31,713,024	0.69	1.60	27.5–28.7	31.1–32.3	-0.24 to -0.23	0.06–0.09
Arahy.13	126,828,166	127,788,471	0.96	0.50	5.7–5.9	6.4–6.6	-0.12 to -0.11	0.02–0.02
Arahy.18	7,198,124	7,347,232	0.15	2.83	13.5–13.6	16.7–16.8	0.15–0.16	0.09–0.10

**Fig. 4:**
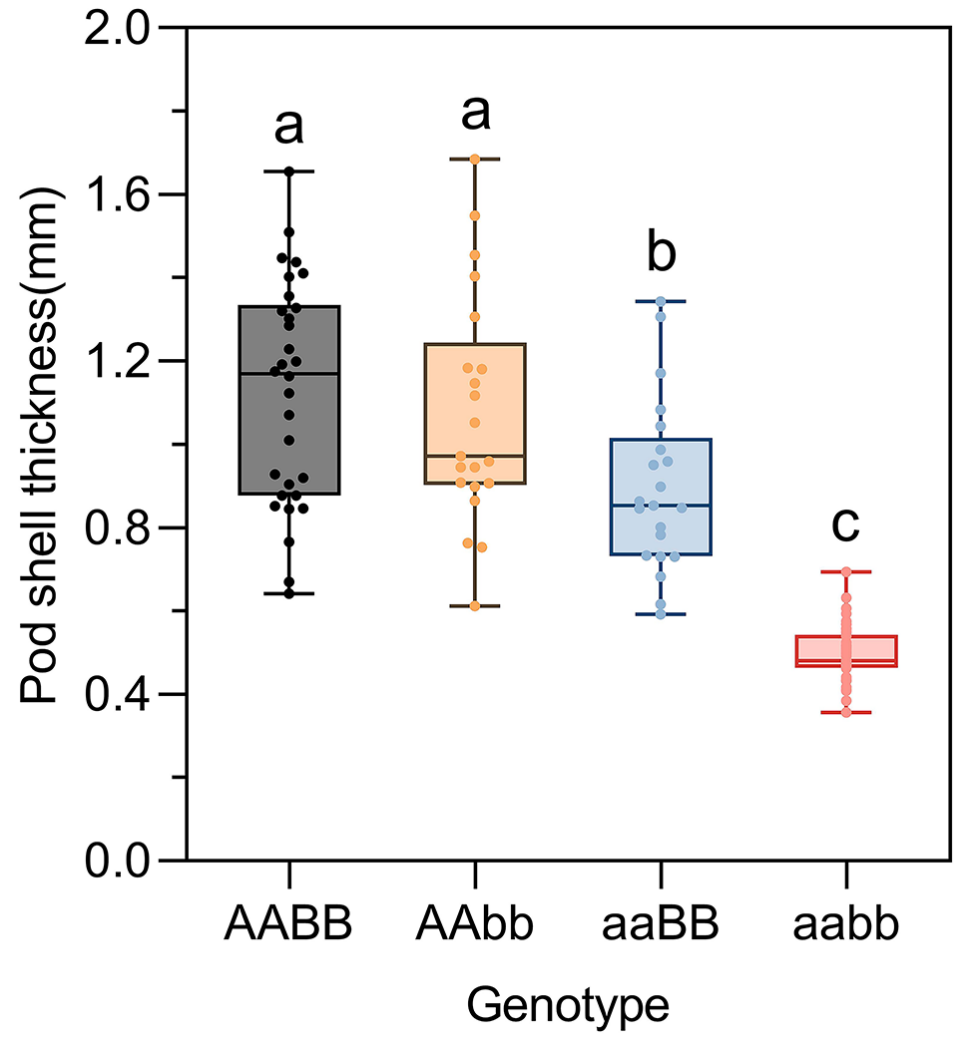
Linkage analysis of the pod shell thickness involving two loci (Tif2_A08_31713024 and Tif2_A18_7198124) in the 410 individuals. Linkage analysis of the pod shell thickness in the 410 individuals of the F_2_ population involving two loci (Tif2_A08_31713024 and Tif2_A18_7198124). Letters indicate significant differences according to a one-way ANOVA/Duncan test (*P* < 0.05)

The 410 individuals of the F_2_ population and the two parents were grouped according to the genotyping results of two markers (Tif2_A08_31713024 and Tif2_A18_7198124). For the Tif2_A08_31713024 locus, the YY18 and WH8 genotypes were denoted as AA and aa, respectively. For the Tif2_A18_7198124 locus, the YY18 and WH8 genotypes were denoted as bb and BB, respectively. Therefore, there were four possible genotypes (AABB, AAbb, aaBB, and aabb) at the two loci. The lines with the AABB genotype had a thick shell, whereas the lines with the aabb genotype had a thin shell. The multiple comparisons test results indicated that the difference in PST between the two genotypes was significant, implying that the genotypes at these two loci were highly correlated with PST (Fig. 4).

### KASP marker validation

**Fig. 5:**
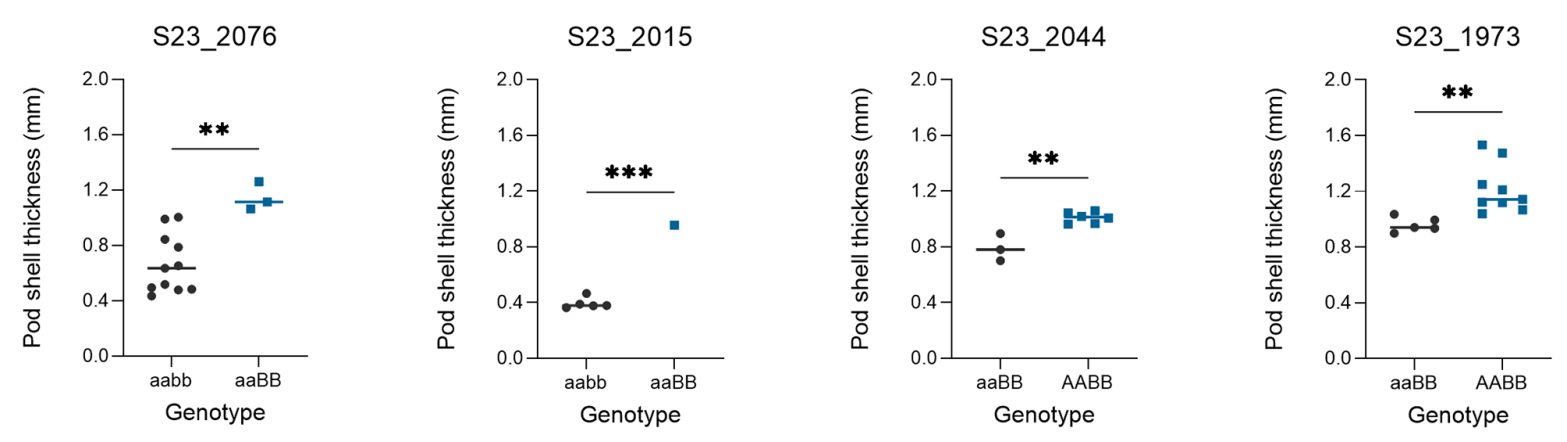
Linkage analysis of the pod shell thickness involving Tif2_A08_31713024 and Tif2_A18_7198124 in four F_2:3_ lines

After genotyping all the F_2_ individuals using the two markers Tif2_A08_31713024 and Tif2_A18_7198124, four individuals were selected from F_2_ to self-cross in May 2023, of which two lines genotyped aaBb, were homozygous at the A08_31713024, while heterozygous at the A18_7198124, two lines genotyped AaBB, were homozygous at the A08_31713024, while heterozygous at the A18_7198124. Linkage analysis of the pod shell thickness involving two loci in four F_2:3_ lines showed that the two markers were associated with PST significantly (Fig. 5).

### Candidate gene annotation

A total of 25 genes were predicted in the candidate regions. According to the resequencing data for the two parents, these genes contained SNPs/InDels. Among these genes, 10, 5, and 10 were detected in the *qPSTA08*, *qPSTA18*, and *qPSTA13* regions, respectively (Table S5). Eight genes had SNPs in the exon regions, resulting in one codon mutation, one frameshift mutation, two synonymous mutations, one missense mutation and three in the untranslated region. Three genes with a nonsynonymous mutation or in the untranslated regions in the two major QTLs (*qPSTA08* and *qPSTA18*), namely *Arahy.R07MUD*, *Arahy.X1RCBJ*, and *Arahy.QT7BNH*, were designated as candidate genes (Table 4).

**Table 4:** The candidate genes with SNPs in the exon regions covered by the three candidate regions

Chromosome	Position (bp)	Gene ID	Annotation	Base variation	Location	Mutation type	Amino acid variation
Arahy.08	31,209,749 − 31,222,270	Arahy.R07MUD	Serine/threonine protein phosphatase family protein	CT-C	3’ UTR	-	-
Arahy.08	31,435,599 − 31,436,846	Arahy.RPFA7S	allene oxide synthase	C-G	CDS	Synonymous mutation	P-P
Arahy.13	126,948,863 − 126,951,270	Arahy.246LCZ	Cytochrome P450 superfamily protein	A-ATCT	CDS	Codon mutation	-S
Arahy.13	127,046,261 − 127,050,581	Arahy.N0D15P	RING/FYVE/PHD zinc finger superfamily protein	TA-T	CDS	Frameshift mutation	-
Arahy.13	127,447,669 − 127,458,789	Arahy.K33YLS	aspartate aminotransferase	A-G	3’ UTR	-	-
Arahy.13	127,490,322 − 127,494,306	Arahy.23HYFJ	myo-inositol-1-phosphate synthase 3	C-T	CDS	Synonymous mutation	L-L
Arahy.18	7,213,210-7,221,785	Arahy.X1RCBJ	ubiquitin carboxyl-terminal hydrolase	G-A	CDS	Missense mutation	M-I
Arahy.18	7,267,302-7,270,853	Arahy.QT7BNH	FASCICLIN-like arabinogalactan protein 16 precursor	G-A	3’ UTR	-	-

## Discussion

### Accurate method for measuring mature peanut pod shell thickness

Previous studies on peanut shells mainly focused on chemical compositions, with relatively little research on the genetic basis of shell traits, especially thickness. Pod shell thickness is usually determined by measuring a fixed position of the shell using a digital vernier caliper. However, because the pod shell has an irregular shape and an uneven thickness, measuring PST at a specific position is inappropriate. Moreover, the shell may deform to some extent if it is examined using vernier calipers and the shell positions used for measuring PST cannot be located precisely. Accordingly, traditional PST measurements may be inaccurate. Therefore, an algorithm was developed to calculate the thickness of the whole pod shell. Specifically, one point on the exocarp was selected and then the closest point on the mesocarp was detected using the algorithm. The distance between the two points was the thickness at that point. After measuring all sampling points on the outer shell, the average value was calculated to determine PST [28]. The number of sampling points (approximately 2,000) was positively correlated with PP. The code of MATLAB algorithm was in supplementary text. Finally, this method resulted in a PST with good repeatability, which contributed to the identification of two major QTLs for PST. Hence, the method developed in this study can accurately measure PST in peanut, with potential implications for measuring the shell thickness of other crops.

### QTLs for traits that are positively or negatively correlated tended to co-localize in the same or adjacent genomic regions

Earlier research indicated PST is strongly positively correlated with PA, PL, and PW [10]. In the current study, the correlation analysis of 350 individuals revealed the highly positive correlations among PST, PA, PL, and PW, but these traits were only weakly positively correlated with PP. Notably, for the two mixed pools, the pods with thick shells were significantly bigger than the pods with thin shells (Figure S2). Therefore, using 22 KASP markers, eight QTLs related to PA, PP, PW, and PL were detected in the same or adjacent regions as the QTLs for PST (Table S6). The QTL (*qSPA08*) for shelling percentage was detected in a region adjacent to *qPSTA08* on chromosome 8 [29]. Besides, Khedikar et al. also identified the QTL for shelling percentage at 43.51 cM within TC6H03-GM1760 on chromosome A08, which may overlap with *qPSTA08* [30]. Furthermore, the PA, PP, PW, and PL values were significantly higher for the samples with the AABB genotype than for the samples with the aabb genotype (Figure S3), suggesting the two major QTLs may be associated with an increase in peanut yield. In previous study, a QTL associated with pod weight and size on chromosome A08 was identified in three consecutive years with 3.33–5.58% PVE [31], which had an overlap with *qPSTA08*. Miao et al. identified QTLs related to pod and seed traits on chromosomes A04, A08, B04, B05, B06 and B08, of which B08 was a major QTL, and two QTL clusters were found on A08 [32, 33].

### Genetic mechanism controlling shell thickness in other crops

The peanut shell develops from the ovary wall and PST may be mainly related to the secondary cell wall (SCW), which is between the plasma membrane and the primary cell wall and is mainly composed of cellulose, hemicellulose, and lignin. Some genes controlling shell thickness in other crops have been identified. In walnut, *JrPXC1* encodes a leucine-rich repeat protein kinase that affects SCW synthesis and functions in xylem fibers [34, 35]. The ortholog of *PXCL* in soybean (*GmLPK1*) is also involved in the development of the cell wall architecture. In an earlier study involving sequencing-based homozygosity mapping, Singh et al. identified the gene controlling oil palm seed shell thickness (*Shell*) and determined it is a homolog of the MADS-box gene *STK* that controls ovary formation and seed development in Arabidopsis [36]. A homologous gene in peach contributes to lignified split-pit formation [37]. In pumpkin, *CpKST1* with a natural mutation controls the hull-less trait by arresting SCW biosynthesis [38]. Other studies indicated NST1 influences anther dehiscence and pod shattering by regulating SCW synthesis in Arabidopsis [39, 40].

### Analysis of candidate genes controlling pod shell thickness in peanut

In the current study, three genes with nonsynonymous mutations in the two major QTLs *qPSTA08* and *qPSTA18*, namely *Arahy.R07MUD*, *Arahy.X1RCBJ*, and *Arahy.QT7BNH*, were predicted as the candidate genes. Of these genes, *Arahy.R07MUD* encodes a serine/threonine protein phosphatase, which is an enzyme that catalyzes the dephosphorylation of the phosphoserine/phosphothreonine side chains of proteins. Serine/threonine protein phosphatases have crucial roles in signal transduction pathways (e.g., MAPK cascades), while also regulating cell cycle-related cell growth and metabolism as well as stress responses and defense activities [41, 42]. In contrast, *Arahy.X1RCBJ* encodes a ubiquitin carboxyl-terminal hydrolase (UCH), which can specifically cleave ubiquitin from proteins during ubiquitin-associated proteasome degradation to facilitate ubiquitin reuse and recycling. The UCHs are a subclass of the deubiquitylating enzymes, which are essential for normal cell metabolism, growth, and development and are involved in important biological processes, including DNA repair and cell apoptosis [43, 44]. The Arabidopsis *uch1* and *uch2* mutants reportedly have altered shoot architecture because the corresponding UCHs directly affect auxin signaling [45]. Finally, *Arahy.QT7BNH* encodes fasciclin-like arabinogalactan protein 16 (FLA16), which helps mediate plant growth and development and cell wall synthesis. Many studies have demonstrated that FLAs are related to cell wall biosynthesis [46–50]. Previous research indicated *FLA16* is mainly expressed in inflorescence tissue cells with secondary cell walls, with the encoded protein playing an important role in plant SCW synthesis and function [51]. Thus, the candidate gene regions will need to be more finely mapped and the candidate genes should be functionally characterized.

## Conclusion

Two major QTLs (*qPSTA08* and *qPSTA18*) for PST were identified on the basis of a BSA-seq analysis and fine mapping. Two molecular markers (Tif2_A08_31713024 and Tif2_A18_7198124) were developed and revealed to be closely linked to PST. The three candidate genes (*Arahy.R07MUD*, *Arahy.X1RCBJ*, and *Arahy.QT7BNH*) were detected in QTL regions.

## Materials and methods

### Plant materials

An F_2_ segregating population consisting of 1,153 individuals was derived from a cross between YY18 (female parent) and WH8 (male parent). Specifically, YY18 is a Spanish-type cultivar that was released in 2015 by the Crops Research Institute, Guangdong Academy of Agricultural Sciences, whereas WH8 is a Virginia-type cultivar that was released in 2003 by the Weifang Academy of Agricultural Sciences. The PST of YY18 (1.17 mm) is greater than that of WH8 (0.60 mm) (Fig. 1A).

### Field trials and phenotyping

The F_2_ population and the two parents were grown in rows at the experimental farm of Henan Academy of Agricultural Sciences, Xinxiang (Henan province) in May 2022, and the F2:3 lines in May 2023, with 0.2 m between plants and 0.4 m between rows. Routine field management practices for peanut production were employed according to local conditions.

Six full and uniform pods were collected from each plant and peeled along the middlemost ridge. The peanut shells were scanned using the Microtek ScanMaker i600 system. Next, the peanut shell exocarp and mesocarp were cut out using Lasso Tool (Photoshop 2020) [52] and a MATLAB algorithm was developed to calculate the average thickness of a whole pod shell (Figure S1). Additionally, PA, PP, PL, and PW were measured using the Image J Fiji software [53].

### Statistical analysis of the phenotypic data

The descriptive statistics analysis, correlation analysis, one-way analysis of viarance, Student’s *t*-test, and multiple comparisons test were performed using the SPSS (version 26.0) software. The frequency distributions of the pod-related traits were visualized using Origin 2022. Histograms and boxplots were created using GraphPad Prism 9.5. The broad-sense heritability was estimated with QTL Icimapping [54].

### Bulk construction, whole-genome resequencing, and SNP calling

Following a visual evaluation, 100 thick-shelled individuals and 100 thin-shelled individuals were selected from 1,153 lines and then analyzed using the MATLAB algorithm. A total of 60 extremely thick-shelled and 60 extremely thin-shelled individuals were screened for the BSA-seq analysis (Fig. 1E). Young leaves were collected from the 120 samples with extreme phenotypes and the two parents and then genomic DNA was extracted using the Plant Genomic DNA Kit (DP305-03; Tiangen). Equal amounts of the genomic DNA extracted from extreme-phenotype materials were mixed to generate the thick-shelled and thin-shelled genomic DNA pools. The two mixed pools and the parental genomic DNA were used for the whole-genome resequencing, which was completed using the Illumina HiSeq™ 2000/MiSeq™ platform to generate 150-bp paired-end reads. The sequencing depths of the mixed pools and the parental genomic DNA were 60× and 30×, respectively.

A CASAVA Base Calling analysis was performed to transform the raw image data obtained from the high-throughput sequencing into raw sequencing reads. During the quality control step, the reads with more than 10% unknown bases or more than 50% low-quality bases (Q ≤ 5) were eliminated [55, 56]. The clean reads were aligned to the *Arachis hypogaea* cv. Tifrunner (version 2) reference genome (https://www.peanutbase.org/). The GATK software [57] was used to detect variants (SNPs and InDels), whereas the SnpEff software [58] was used to annotate variants and predict variant effects.

### BSA-seq analysis

In this study, Δ(SNP-index), ED, *G* statistics, and *P* values were used to identify the SNPs associated with PST, with a 2-Mb sliding window and a step size of 10 kb (https://github.com/xiekunwhy/bsa). A 99% confidence level was selected as the threshold. The overlapping intervals among the four methods were considered as the candidate QTL regions associated with PST. The genes in the candidate intervals were annotated using an online resource (https://www.peanutbase.org/).

### KASP marker development, QTL validation, and fine mapping

To narrow the candidate intervals, 410 materials comprising 120 extreme-phenotype individuals and 290 randomly selected individuals from the F_2_ population were used for data validation and fine mapping. As previously described, the resequencing data of the two parents were used to convert the polymorphic SNPs in the candidate intervals to KASP markers [59]. For each KASP marker, two allele-specific forward primers and one common reverse primer were designed according to the 200 bp upstream and downstream genomic sequences flanking the genic SNPs [60].

Joinmap (version 5.0) [61] was used to construct the genetic linkage map on the basis of independent LOD scores ranging from 2 to 28. Loci orders were determined using the maximum likelihood algorithm and Kosambi’s mapping function. In addition, multiple QTL mapping (MQM) of MapQTL 6 [62] was used for analyzing QTLs. The LOD threshold was set as 2.5, with a mapping step size of 0.1 cM. The QTL regions were drawn using MapChart 2.3 [63].

### Electronic supplementary material

Below is the link to the electronic supplementary material.

Supplementary Material 1. **Addition file 1: Supplementary Tables S1-S6.Table S1** The information of the 410 individuals of the F2 population. **Table S2** The descriptive statistics of two parents and the 410 individuals of the F2 population. **Table S3** The one-way ANOVA of the 410 individuals of the F2 population. **Table S4.** KASP markers used in this study. **Table S5.** Genes with SNPs between the two parents in the three candidate regions. **Table S6.** QTLs identified for pod size-related traits in the 350 individuals of F_2_ population.

Supplementary Material 2. **Fig. S1.** Process for calculating the pod shell thickness using the MATLAB algorithm. (A) Image produced by a scanner. (B) Image of the peanut shell exocarp and mesocarp cut out using Lasso Tool. (C) Image of the peanut shell part (red outline) used to calculate PST according to the MATLAB algorithm. **Fig. S2.** Pod-related traits of the thick-shelled pool and thin-shelled pool. Pod shell thickness (A), pod area (B), pod perimeter (C), pod width (D), and pod length (E) of the thin-shelled pool and thick-shelled pool. ***, *P* < 0.001. **Fig. S3.** Linkage analysis of the pod-related traits involving two loci (Tif2_A08_31713024 and Tif2_A18_7198124). Linkage analysis of the pod area (A), pod perimeter (B), pod width (C), and pod length (D) of the 350 individuals in the F2 population involving two loci (Tif2_A08_31713024 and Tif2_A18_7198124). Letters indicate significant differences according to a one-way ANOVA/Duncan test (*P* < 0.05). **Fig. S4.** The correlation between the two methods in the 88 thick individuals. Horizontal coordinates represent the PST measured at the waists by vernier calipers. Vertical coordinates represent the PST measured by MATLAB algorithm.

Supplementary Material 3. Supplementary Text. The code for the MATLAB algorithm for culculaing the pod shell thickness.

## Data Availability

All data generated or analyzed during this study are included in the manuscript and its Additional file 1 to Additional file 6. The clean data of the two parents and two bulked pools obtained in this study have been submitted to the BioProject database at NCBI under the BioProject ID: PRJCA019117. The materials used during the current study are available from the corresponding authors.

## Abbreviations

PST: Pod Shell Thickness
QTLs: Quantitative Trait Loci
BSA-seq: Bulked Sergeant Analysis Sequencing
KASP: Kompetitive Allele-specific PCR
PVE: Phenotypic Variance Explained
SNPs: Single Nucleotide Polymorphisms
Indels: Insertion-deletion
PA: Pod area
PP: Pod Perimeter
PL: Pod length
PW: Pod width
SCW: Secondary Cell Wall

## References

[1] Güzel E, Akcali I, Mutlu H, Ince A (2005). Research on the fatigue behavior for peanut shelling. J Food Eng.

[2] Guzman JD, Petingco MC, Dom-oguen ADP. Peanut threshing and shelling machines for community-based peanut enterprises in developing countries, in ASABE Annual International Meeting. Am Soc Agri Bio Eng; 2019. p. 1.

[3] Cui K, Qi F, Sun Z, Feng J, Huang B, Dong W, Zhang X (2021). Genome-wide association study of physical and microstructure-related traits in peanut shell. Plant Genet Resour.

[4] Ding B, Xie J, Feng S, Chen Z, Jiang Y (2020). Effect of different pod types on mechanical husking of peanut. J Jiangsu Agric Sci.

[5] Wee J-H, Moon J-H, Eun J-B, Chung J-H, Kim Y-G, Park K-H (2007). Isolation and identification of antioxidants from peanut shells and the relationship between structure and antioxidant activity. Food Sci Biotechnol.

[6] Guo H. Construction of chromosome segment substitution lines and QTLs mapping for agronomic traits in cultivated peanut. Master Thesis, Hebei Agriculture University, China, 2014 (in Chinese with English abstract).

[7] Li Y. QTL analysis for height, total branching number and pod traits in peanut (Arachis hypogaes L.). Master Thesis, Shandong Agriculture University, China, 2016 (in Chinese with English abstract).

[8] Liu J. Mapping and analysis of QTLs for agronomic and quality traits using RIL population of Shanhua15×Zhonghua12 in peanut (Arachis hypogaes L.). Master Thesis, Shandong Agriculture University, China, 2017 (in Chinese with English abstract).

[9] Yang Q. High density genetic linkage map construction and QTL mapping for pods size related traits in peanut (Arachis hypogaes L.). Master Thesis, Fujian Agriculture and Forestry University, China, 2018 (in Chinese with English abstract).

[10] Yang H, Luo L, Li Y, Li H, Zhang X, Zhang K, Zhu S, Li X, Li Y, Wan Y (2023). Wan. Fine mapping of qAHPS07 and functional studies of AhRUVBL2 controlling pod size in peanut (Arachis hypogaea L). Plant Biotechnol J.

[11] Li Z, Xu Y (2022). Bulk segregation analysis in the NGS era: a review of its teenage years. Plant J.

[12] Li C, Ling F, Su G, Sun W, Liu H, Su Y, Qi X (2020). Location and mapping of the NCLB resistance genes in maize by bulked segregant analysis (BSA) using whole genome re-sequencing. Mol Breed.

[13] SU B-H, ZHOU M-M, LIU Z-M OCHARK, GAO H-W, Lamlom SF (2022). QIU L-J. Identification of the genetic locus associated with the crinkled leaf phenotype in a soybean (Glycine max L.) mutant by BSA-Seq technology. J Integr Agr.

[14] Sun J, Wang J, Guo W, Yin T, Zhang S, Wang L, Xie D, Zou D (2021). Identification of alkali-tolerant candidate genes using the NGS-assisted BSA strategy in rice. Mol Breed.

[15] Klymiuk V, Chawla HS, Wiebe K, Ens J, Fatiukha A, Govta L, Fahima T, Pozniak CJ (2022). Discovery of stripe rust resistance with incomplete dominance in wild emmer wheat using bulked segregant analysis sequencing. Commun Biol.

[16] Zhang K, Yuan M, Xia H, He L, Ma J, Wang M, Zhao H, Hou L, Zhao S, Li P (2022). BSA–seq and genetic mapping reveals AhRt2 as a candidate gene responsible for red testa of peanut. Theor Appl Genet.

[17] Pan J, Zhou X, Ahmad N, Zhang K, Tang R, Zhao H, Jiang J, Tian M, Li C, Li A (2022). BSA–seq and genetic mapping identified candidate genes for branching habit in peanut. Theor Appl Genet.

[18] Guo J, Qi F, Qin L, Zhang M, Sun Z, Li H, Cui M, Zhang M, Li C, Li X. Mapping of a QTL associated with sucrose content in peanut kernels using BSA-seq. Front Genet 2022, 13. 10.3389/fgene.2022.1089389 PMC9845247 36685909

[19] Takagi H, Abe A, Yoshida K, Kosugi S, Natsume S, Mitsuoka C, Uemura A, Utsushi H, Tamiru M, Takuno S (2013). QTL-seq: rapid mapping of quantitative trait loci in rice by whole genome resequencing of DNA from two bulked populations. Plant J.

[20] Hill JT, Demarest BL, Bisgrove BW, Gorsi B, Su Y-C, Yost HJ (2013). MMAPPR: mutation mapping analysis pipeline for pooled RNA-seq. Genome Res.

[21] Magwene PM, Willis JH, Kelly JK (2011). The statistics of bulk segregant analysis using next generation sequencing. Plos Comput Biol.

[22] Wang C, Tang S, Zhan Q, Hou Q, Zhao Y, Zhao Q, Feng Q, Zhou C, Lyu D, Cui L (2019). Dissecting a heterotic gene through GradedPool-Seq mapping informs a rice-improvement strategy. Nat Commun.

[23] Broccanello C, Chiodi C, Funk A, McGrath JM, Panella L, Stevanato P (2018). Comparison of three PCR-based assays for SNP genotyping in plants. Plant Methods.

[24] Majeed U, Darwish E, Rehman SU, Zhang X (2018). Kompetitive allele specific PCR (KASP): a singleplex genotyping platform and its application. J Agri Sci.

[25] Xin W, Liu H, Yang L, Ma T, Wang J, Zheng H, Liu W, Zou D (2022). BSA-Seq and fine linkage mapping for the identification of a novel locus (qPH9) for mature plant height in rice (Oryza sativa). Rice.

[26] Cao Y, Diao Q, Chen Y, Jin H, Zhang Y, Zhang H (2021). Development of KASP markers and identification of a QTL underlying powdery mildew resistance in melon (Cucumis melo L.) by bulked segregant analysis and RNA-seq. Front Plant Sci.

[27] Xie X, Li S, Liu H, Xu Q, Tang H, Mu Y, Deng M, Jiang Q, Chen G, Qi P (2022). Identification and validation of a major QTL for kernel length in bread wheat based on two F3 biparental populations. BMC Genomics.

[28] Wang W, Ji T (2018). Adaptive analysis method for particles image. Multimed Tools Appl.

[29] Li W, Liu N, Huang L, Chen Y, Guo J, Yu B, Luo H, Zhou X, Huai D, Chen W (2022). Stable major QTL on chromosomes A07 and A08 increase shelling percentage in peanut (Arachis hypogaea L). Crop J.

[30] Khedikar Y, Pandey MK, Sujay V, Singh S, NS N, Klein-Gebbinck HW, Cholin S, Mukri G, Garg V, Upadhyaya HD (2018). Identification of main-effect and epistatic quantitative trait loci for morphological and yield related traits in peanut (Arachis hypogaea L). Mol Breed.

[31] Luo H, Guo J, Ren X (2018). Chromosomes A07 and A05 associated with stable and major QTLs for pod weight and size in cultivated peanut (*Arachis hypogaea* L). Theor Appl Genet.

[32] Miao P, Meng X, Li Z, Sun S, Chen CY, Yang X (2023). Mapping quantitative trait loci (QTLs) for hundred-pod and hundred-seed weight under seven environments in a recombinant inbred line Population of cultivated peanut (*Arachis hypogaea* L). Genes (Basel).

[33] Meng X, Zhang J, Cui S, Charles Y, Mu G, Hou M, Yang L, Liu L (2021). QTL mapping and QTL × environment interaction analysis of pod and seed related traits in cultivated peanut (Arachis hypogaea L). The crop J.

[34] Barra-Jiménez A, Ragni L (2017). Secondary development in the stem: when Arabidopsis and trees are closer than it seems. Curr Opin Plant Biol.

[35] Wang J, Kucukoglu M, Zhang L, Chen P, Decker D, Nilsson O, Jones B, Sandberg G, Zheng B (2013). The Arabidopsis LRR-RLK, PXC1, is a regulator of secondary wall formation correlated with the TDIF-PXY/TDR-WOX4 signaling pathway. BMC Plant Biol.

[36] Singh R, Low E-TL, Ooi LC-L, Ong-Abdullah M, Ting N-C, Nagappan J, Nookiah R, Amiruddin MD, Rosli R, Manaf MAA (2013). The oil palm SHELL gene controls oil yield and encodes a homologue of SEEDSTICK. Nature.

[37] Tani E, Polidoros AN, Flemetakis E, Stedel C, Kalloniati C, Demetriou K, Katinakis P, Tsaftaris AS (2009). Characterization and expression analysis of AGAMOUS-like, SEEDSTICK-like, and SEPALLATA-like MADS-box genes in peach (Prunus persica) fruit. Plant Physiol Bioch.

[38] Lyu X, Shi L, Zhao M, Li Z, Liao N, Meng Y, Ma Y, Zhou Y, Xue Q, Hu Z (2022). A natural mutation of the NST1 gene arrests secondary cell wall biosynthesis in the seed coat of a hull-less pumpkin accession. Hortic Res.

[39] Mitsuda N, Seki M, Shinozaki K, Ohme-Takagi M (2005). The NAC transcription factors NST1 and NST2 of Arabidopsis regulate secondary wall thickenings and are required for anther dehiscence. Plant Cell.

[40] Mitsuda N, Ohme-Takagi M (2008). NAC transcription factors NST1 and NST3 regulate pod shattering in a partially redundant manner by promoting secondary wall formation after the establishment of tissue identity. Plant J.

[41] Mumby MC, Walter G (1993). Protein serine/threonine phosphatases: structure, regulation, and functions in cell growth. Physio Rev.

[42] Shi Y (2009). Serine/threonine phosphatases: mechanism through structure. Cell.

[43] Hayama R, Yang P, Valverde F, Mizoguchi T, Furutani-Hayama I, Vierstra RD, Coupland G (2019). Ubiquitin carboxyl-terminal hydrolases are required for period maintenance of the circadian clock at high temperature in Arabidopsis. Sci Rep.

[44] Wang D-H, Song W, Wei S-W, Zheng Y-F, Chen Z-S, Han J-D, Zhang H-T, Luo J-C, Qin Y-M, Xu Z-H (2018). Characterization of the ubiquitin C-terminal hydrolase and ubiquitin-specific protease families in rice (Oryza sativa). Front Plant Sci.

[45] Yang P, Smalle J, Lee S, Yan N, Emborg TJ, Vierstra RD (2007). Ubiquitin C-terminal hydrolases 1 and 2 affect shoot architecture in Arabidopsis. Plant J.

[46] MacMillan CP, Mansfield SD, Stachurski ZH, Evans R, Southerton SG (2010). Fasciclin-like arabinogalactan proteins: specialization for stem biomechanics and cell wall architecture in Arabidopsis and Eucalyptus. Plant J.

[47] Shi H, Kim Y, Guo Y, Stevenson B, Zhu J-K (2003). The Arabidopsis SOS5 locus encodes a putative cell surface adhesion protein and is required for normal cell expansion. Plant Cell.

[48] Wang H, Jiang C, Wang C, Yang Y, Yang L, Gao X, Zhang H (2015). Antisense expression of the fasciclin-like arabinogalactan protein FLA6 gene in Populus inhibits expression of its homologous genes and alters stem biomechanics and cell wall composition in transgenic trees. J Exp Bot.

[49] Wang H, Jin Y, Wang C, Li B, Jiang C, Sun Z, Zhang Z, Kong F, Zhang H (2017). Fasciclin-like arabinogalactan proteins, PtFLAs, play important roles in GA-mediated tension wood formation in Populus. Sci Rep.

[50] Ito S, Suzuki Y, Miyamoto K, Ueda J, Yamaguchi I (2005). AtFLA11, a fasciclin-like arabinogalactan-protein, specifically localized in screlenchyma cells. Biosci Biotech Bioch.

[51] Liu E, MacMillan CP, Shafee T, Ma Y, Ratcliffe J, Van de Meene A, Bacic A, Humphries J, Johnson KL (2020). Fasciclin-like arabinogalactan-protein 16 (FLA16) is required for stem development in Arabidopsis. Front Plant Sci.

[52] Park JS, Chung MS, Hwang SB, Lee YS, Har D-H. Technical report on semiautomatic segmentation using the Adobe Photoshop. J digit imaging. 2005;18:333–343. 10.1007/s10278-005-6704-1 PMC3046725 16003588

[53] Hartmann A, Czauderna T, Hoffmann R, Stein N, Schreiber F (2011). HTPheno: an image analysis pipeline for high-throughput plant phenotyping. BMC Bioinformatics.

[54] Meng L, Li H, Zhang L, Wang J (2015). QTL IciMapping: Integrated software for genetic linkage map construction and quantitative trait locus mapping in biparental populations. The crop J.

[55] Peter, Cock C, Fields N, Goto M (2010). The Sanger FASTQ file format for sequences with quality scores, and the Solexa/Illumina FASTQ variants. Nucleic Acids Res.

[56] Hansen KD, Brenner SE, Sandrine D (2010). Biases in Illumina transcriptome sequencing caused by random hexamer priming. Nucleic Acids Res.

[57] McKenna A, Hanna M, Banks E, Sivachenko A, Cibulskis K, Kernytsky A, Garimella K, Altshuler D, Gabriel S, Daly M (2010). The genome analysis Toolkit: a MapReduce framework for analyzing next-generation DNA sequencing data. Genome Res.

[58] Cingolani P, Platts A, Wang LL, Coon M, Nguyen T, Wang L, Land SJ, Lu X, Ruden DM (2012). A program for annotating and predicting the effects of single nucleotide polymorphisms, SnpEff: SNPs in the genome of Drosophila melanogaster strain w1118; iso-2; iso-3. Fly.

[59] Trick M, Adamski NM, Mugford SG, Jiang C-C, Febrer M, Uauy C (2012). Combining SNP discovery from next-generation sequencing data with bulked segregant analysis (BSA) to fine-map genes in polyploid wheat. BMC Plant Biol.

[60] He C, Holme J, Anthony J (2014). SNP genotyping: the KASP assay. Methods Mol Bio.

[61] Ooijen V (2018). JoinMap® 5, Software for the calculation of genetic linkage maps in experimental populations of diploid species.

[62] Van Ooijen J, Kyazma B. MapQTL 6, Software for the mapping of quantitative trait loci in experimental populations of diploid species. Kyazma BV: Wageningen, Netherlands. 2009;5.

[63] Voorrips R (2002). MapChart: software for the graphical presentation of linkage maps and QTLs. J Hered.

