# Supplementary material for: Identification of two major QTLs for pod shell thickness in peanut (Arachis hypogaea L.) using BSA-seq analysis
Source: BMC Genomics. 2024 Jan 16;25:65. doi: 10.1186/s12864-024-10005-x (PMC10790476; doi:10.1186/s12864-024-10005-x)
Supplement: Supplementary file 2 — Supplementary Material 2. Fig. S1. Process for calculating the pod shell thickness using the MATLAB algorithm. (A) Image produced by a scanner. (B) Image of the peanut shell exocarp and mesocarp cut out using Lasso Tool. (C) Image of the peanut shell part (red outline) used to calculate PST according to the MATLAB algorithm. Fig. S2. Pod-related traits of the thick-shelled pool and thin-shelled pool. Pod shell thickness (A), pod area (B), pod perimeter (C), pod width (D), and pod length (E) of the thin-shelled pool and thick-shelled pool. ***, P < 0.001. Fig. S3. Linkage analysis of the pod-related traits involving two loci (Tif2_A08_31713024 and Tif2_A18_7198124). Linkage analysis of the pod area (A), pod perimeter (B), pod width (C), and pod length (D) of the 350 individuals in the F2 population involving two loci (Tif2_A08_31713024 and Tif2_A18_7198124). Letters indicate significant differences according to a one-way ANOVA/Duncan test (P < 0.05). Fig. S4. The correlation between the two methods in the 88 thick individuals. Horizontal coordinates represent the PST measured at the waists by vernier calipers. Vertical coordinates represent the PST measured by MATLAB algorithm. [file 12864_2024_10005_MOESM2_ESM.docx]

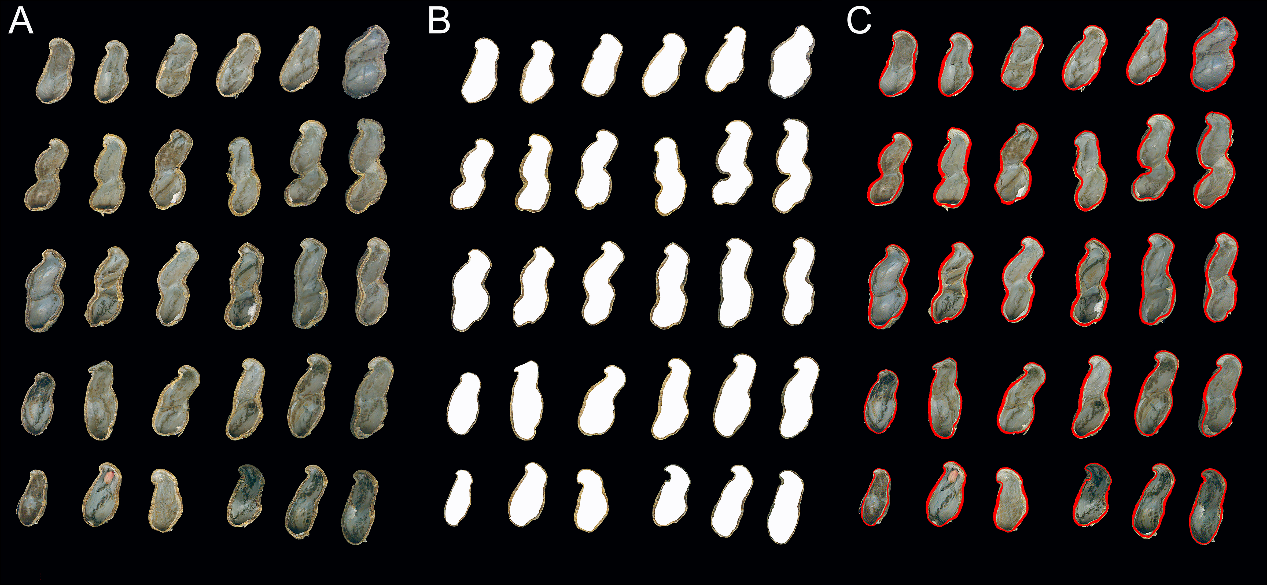


**Fig. S1.** Process for calculating the pod shell thickness using the MATLAB algorithm. (A) Image produced by a scanner. (B) Image of the peanut shell exocarp and mesocarp cut out using Lasso Tool. (C) Image of the peanut shell part (red outline) used to calculate PST according to the MATLAB algorithm.


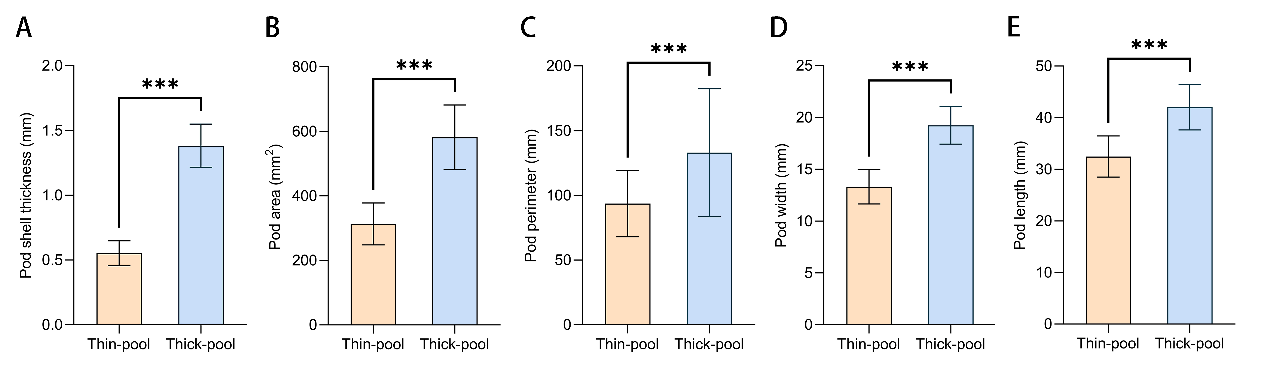


**Fig. S2.** Pod-related traits of the thick-shelled pool and thin-shelled pool. Pod shell thickness (A), pod area (B), pod perimeter (C), pod width (D), and pod length (E) of the thin-shelled pool and thick-shelled pool. ***, *P* < 0.001


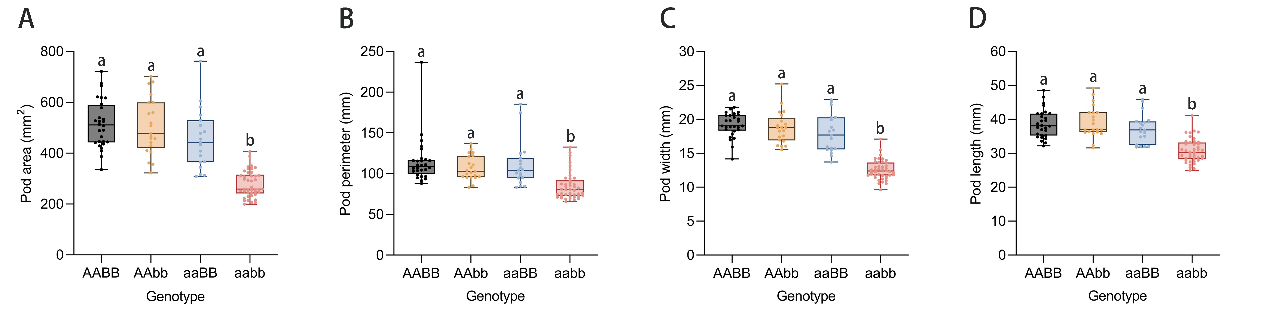


**Fig. S3.** Linkage analysis of the pod-related traits involving two loci (Tif2_A08_31713024 and Tif2_A18_7198124). Linkage analysis of the pod area (A), pod perimeter (B), pod width (C), and pod length (D) of the 350 individuals in the F_2_ population involving two loci (Tif2_A08_31713024 and Tif2_A18_7198124). Letters indicate significant differences according to a one-way ANOVA/Duncan test (*P* < 0.05).


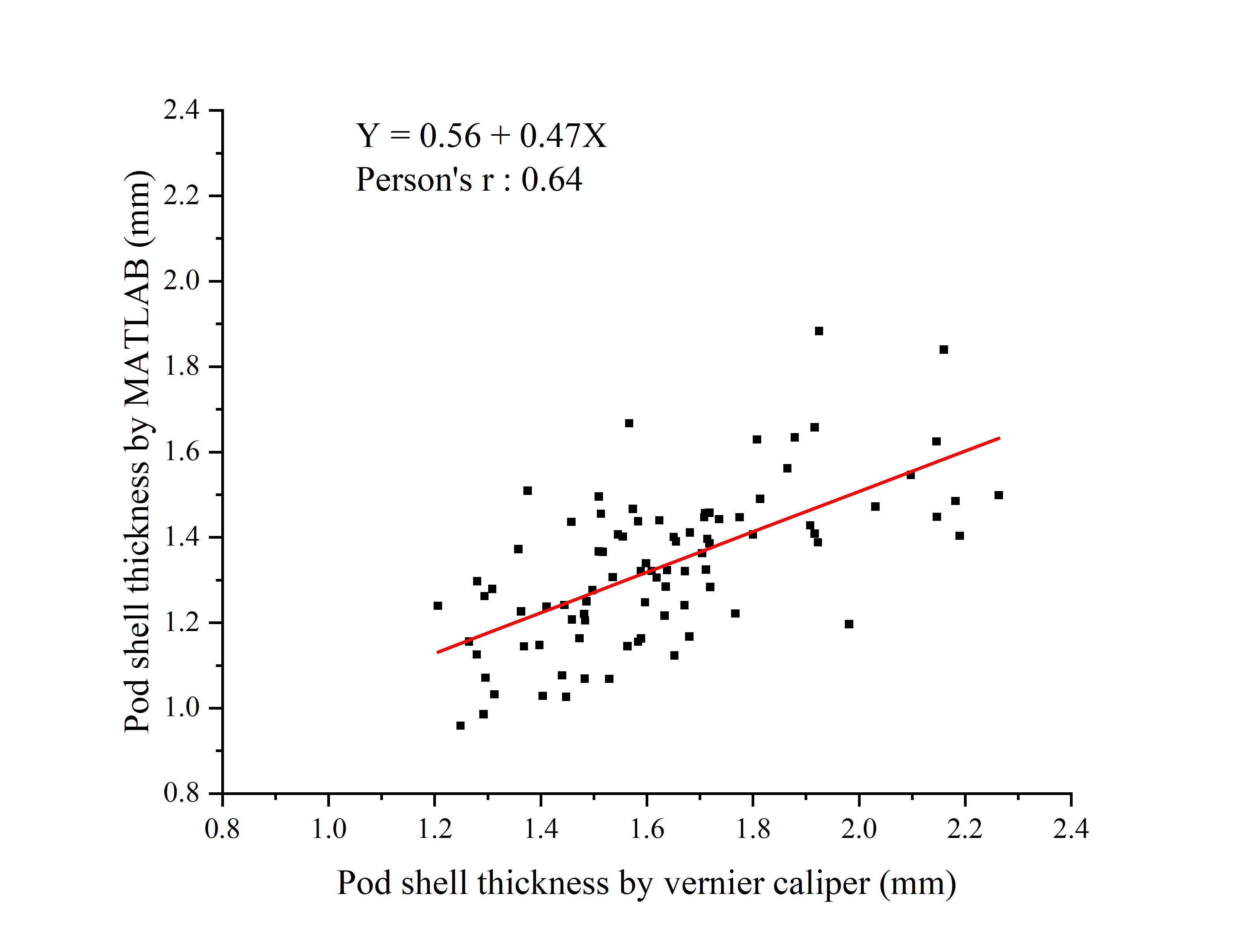


**Fig. S4.** The correlation between the two methods in the 88 thick individuals. Horizontal coordinates represent the PST measured at the waists by vernier calipers. Vertical coordinates represent the PST measured by MATLAB algorithm.
